# As the bell tolls: a foundation study on pancreatic cancer consumer's research priorities

**DOI:** 10.1186/1756-0500-2-179

**Published:** 2009-09-09

**Authors:** Carla Saunders, Helen Gooden, Monica Robotin, Jan Mumford

**Affiliations:** 1 School of Medicine and Public Health, The University of Newcastle, Wallsend NSW, Australia; 2 The Cancer Council NSW, 153 Dowling Street, Woolloomooloo, NSW Australia; 3 Faculty of Nursing & Midwifery, University of Sydney, Camperdown NSW, Australia; 4 School of Public Health University of Sydney, Camperdown NSW, Australia

## Abstract

**Background:**

This is the first investigation of its kind to explore the views of people affected by pancreatic cancer with regard to research priorities. Pancreatic cancer has an extremely poor outlook in terms of early diagnosis, effective treatment and survival. Those affected by the disease generally lack opportunities to voice their needs or concerns in an organised manner, link with others affected by the condition and take part in research.

**Methods:**

This qualitative study adopts a self-selected telephone focussed discussion group approach. Information was obtained from distinct carer and patient groups after adequate controls such as the 'safe space' technique (repeatedly enquiring on and respecting the emotional needs) were implemented to protect participants from undue physical and psychological distress.

**Results:**

Five themes emerged overall, with three themes being common between the patients and carers groups. Early detection, clinician communication and public awareness were areas of recurring discussion and consensus for both groups. The fourth theme to emerge for the patient group centred on quality of care, while the fourth theme of the carer group focused on the need for more and improved treatment options.

**Conclusion:**

Research priorities for pancreatic cancer consumers have been identified via an investigation that was tailored to meet exceptional needs. This research gives us a primary understanding of the role that pancreatic cancer patients can play in identifying areas of research that are responsive to their needs and priorities when suitably planned. Importantly it also provides a much greater understanding of the grim realities of the disease for those affected. This work is likely to be of value to anyone planning to work with those with a time limited, challenging condition.

## Background

Pancreatic cancer (PC) is a deadly disease. The early vague symptoms are easily overlooked and high-risk delays in diagnosis are very common. Only around 10% of patients are considered for surgical treatment [1]. PC is the fourth leading cause of death in Western societies and recently the national rate of new cases in Australia has increased by 30% [2]. The ageing of the Australian population means that a proportionate increase in the number of people affected by PC, including carers, can be expected. Currently, few formal protocols exist for best clinical practice in PC management and no multidisciplinary care teams are reported [3].

There has been little improvement in the 5 year PC survival rate over the last few decades which have remained extremely low at around 5% with the majority of patients (~90%) dying within a year of diagnosis [4]. This compares very poorly with relative 5 year survival rates for more common malignancies such as melanoma, breast and prostate cancer which are greater than 80%, and for all cancers combined (65%) [5]. Contributing to this unfortunate situation is the long-standing relative lack of scientific knowledge of the disease; including research in all aspects, from aetiology through to effective palliative care, and an understanding of how those affected endure in daily life.

Research can lead to new or advances in treatment, improved life expectancy, better quality of life, reduced social burden and a wide range of other potential benefits. Until recently, the Australian scientific community, who largely determine priorities for cancer research, had not often selected PC. Dedicated funding programs targeting this cancer and other understudied human diseases have now been developed by a key government research funding body to encourage a more balanced approach in the spread of research across different diseases with the goal of reducing occurrence, morbidity and adverse outcomes [6].

Cancer Council NSW (CCNSW) is NSW's largest publicly funded cancer charity, which supports a significant proportion of Australia's cancer research. In 2006, one of the research grant schemes of CCNSW, the *Strategic Research Partnership Grant *[7], founded a network of key PC researchers and consumers affected by PC. An important function of the *NSW Pancreatic Cancer Network *[8] has been to systematically define critical research issues and opportunities that could accelerate progress in PC research in Australia. The rigorous prioritisation process began with a literature review, followed by structured interviews with key opinion leaders in the field, whose recommendations were then fed into a Delphi consensus process. The recommendations from the Delphi and discrete consumer involvement processes were ultimately prioritised via a nominal group procedure.

The aim of this paper is to describe the technique used to capture consumer priorities in PC research, together with the findings of the investigation. Consumers are recognised as patients and carers of people diagnosed with PC.

Cancer research is a matter of great public interest in NSW [9] and it is very important to CCNSW that both researchers and consumers are involved in research decision making processes [10], as failure to involve the community is likely to result in important areas of inquiry not being recognised. Involving consumers in research prioritisation is still a novel process both in Australia and in other countries, and seeking consumer views on research priorities in PC presents additional challenges, as diagnosis at an advanced stage, rapid disease progression and overt symptoms, including pain, limits the applicability of traditional methods of consumer consultation.

Currently the academic literature tells us that consumers have had dialogue with research funders about research priorities/gaps, they have been involved in the priority ranking of pre-established research questions and as members on committees they have collaborated with scientists to prioritise research across a range of health topics including breast cancer, mental health, physical and complex disabilities, young people, cystic fibrosis and HIV [11-13]. Although less common, consumers have also independently identified issues and set research agendas for specific cancer types [14], nevertheless we have been unable to identify any inquiries that provide those affected by pancreatic cancer the opportunity to interact, discuss and identify research priorities that are important to them.

## Methods

This study was designed in consultation with a key stakeholder group comprising pancreatic cancer researchers, consumers and representatives of CCNSW. The Concord Hospital Human Research Ethics Committee (NSW, Australia) approved the research. Informed consent was gained from all research participants.

Self-selected participants were recruited from CCNSW website and newsletter information which described the opportunities and benefits of involvement in a newly launched *CCNSW Pancreatic Cancer Support Project *which was established to identify and address unmet supportive care needs of PC patients, and current or bereaved carers. Consumers opting into the Project were informed of the opportunity and invited to take part in a focused discussion to identify pancreatic cancer research priorities. Voluntary and informed consent to participate in the research was gained after all relevant information was provided and interested persons assured us they had adequate understanding of the study requirements.

Participants were given the option of a traditional face-to-face discussion or one conducted by telephone, with telephone delivered focussed discussion the preferred option. One discussion group comprised patients with a PC diagnosis while another involved current and bereaved carers of PC patients. Separating the groups allowed for focussed discussion on issues that particularly affected each group.

Other investigators have used teleconference focussed discussions as the major/sole way to collect data, primarily to overcome distance and participant particular needs, and found them to be successful [15-17]. The limitations highlighted in these studies such as the increased difficulty in controlling participants using the phone method were taken into account in the design of the current study.

The focussed discussion method was selected so we could examine not only what PC consumers thought, but also why they thought it via comparing and contrasting personal experiences and perspectives in group discussion. The basic assumption of this interpretive approach was that relevant information would be gained through interaction with others, shared meanings and conclusions.

As the research involved gaining an in-depth understanding of issues, including an exploration of the reasons and context for participants' viewpoints and actions, the facilitators agreed on a 'safe space' approach prior to the focus groups. This approach of repeatedly enquiring on and respecting the emotional needs of participants allowed them to openly disclose (and acknowledge in others) emotional and/or physical distress without fear of disrupting the discussion, particularly when the dialogue centred on suffering and/or death [18,19].

The facilitated discussions were audiotaped and subsequently transcribed. Two CCNSW telephone support group counsellors, one acting as moderator; an independent qualitative researcher, and the Project coordinator, as subject expert, facilitated the group discussions. All participants were provided with the purpose and ground rules, and were assured of the confidential nature of the information gathered during the discussions. The moderator also explained the need for audio-recording the discussion for analysis purposes.

At the commencement of the focussed discussions participants in each group were asked the question: *Where would you like to see progress in pancreatic cancer being made? *After participants raised and discussed each issue they felt was important to the topic, the facilitator fed the main points back to confirm the interpretation of comments and to reach agreement on the main themes of the discussion.

Before ending each group discussion by formally thanking participants, the moderator provided participants with information on the cost free CCNSW professional telephone support services to provide them with an ongoing avenue to speak about their experiences and concerns in the future.

### Data analysis

A thematic content analysis was manually conducted with pattern recognition within the data identified after careful listening to the audiotape and reading/re-reading the transcripts by two researchers (CS and MR) independently. Acknowledging the context and participants of the research, repeating issues were identified and clustered, and subsequently developed into focal themes.

## Results

### Demographics

Twelve people responded to the invitation to participate in the study with eleven taking part after ill health prevented the participation of one male. Five current and bereaved carers made up one group, with one bereaved carer providing email responses to the questions raised with the group. Six people diagnosed with PC took part in the other phone group discussion. Table 1 provides the demographic characteristics of the research participants.

**Table 1:** Demographic Characteristics of the Research Participants

	**Focus Groups (n = 11)**	
	1 (patients)	2 (carers)
Gender		
Male	1	1
Female	5	4
Age ranges (years)		
Male	70-79	60-69
Female	30-59	30-49
Place of Residence		
Urban	4	4
Regional/rural	1	1

Unknown	1	

### Identified themes

The carers and patients discussion groups each lasted approximately one hour. For the most part, discussion was based on personal experiences rather than opinion or conjecture. The audiotapes provided an understanding of the tone of conversations and the levels of urgency particular topics produced. Overall, participants in each group verbally expressed a range of emotions throughout the session from anger and frustration to grief. Most were active information providers and many expressed considerable worry. In general, there was no area of discussion in which each group of participants failed to reach general consensus.

Five themes emerged overall, with three being common between the patients and carers groups. Early detection, clinician communication and public awareness were areas of recurring discussion and consensus for both groups. The fourth theme to emerge for the patient group centred on quality of care, while the fourth theme of the carer group focused on the need for more and improved treatment options.

Tables 2 and 3 provide a complete overview of the identified focal themes and the repeated issues from which they were derived for each group.

**Table 2:** Pancreatic cancer patients repeated issues and focal themes

**Recurring issue**	**Focal theme**
*"I would like to see some work done in the area of symptoms and diagnostic tools so people who have got a problem can hopefully get a quicker result"* *"People that have had gallstones and have had their gall bladder out, and 18 months later it's been diagnosed as pancreatic cancer. So it's those sorts of correlations and the diagnostic tools need to be fine-tuned"* *"I agree with you ... I had to have a battery of tests to sort mine out, basically because I was pretty sick at the time. I would like to see the testing simplified a bit. I was sick for a couple of months before, but I had to be sick enough to be in hospital before it was found"* *"Cancer is the silent killer. By the time I was diagnosed it was too late. "Mine had already moved over to the liver and the spleen and so there was no surgery involved because there were too many organs"* *"Early diagnosis would be ideal. I found out when it was too late*.... *"People need to get to the doctor before it's too late so they can have options"*	Early detection
*"I first heard of my pancreatic cancer on 18 July when I first went in to see the doctor. He sent me straight for an MRI. I hate him for what he said when I came back five hours later: 5 o'clock on a Friday night I'm a healthy person, I'm lively, I had a life planned to 95, and he told me I would be dead in six to 18 months. I was stunned...... I think what I would like to say here is we must have information presented in a professional form"* *"While treatment is happening and I get side effects, the nurses never clarify that it is a side effect. They just send me to my GP and unfortunately my GP is very much like your GP -- I don't get a lot of information. He needs correct information about side effects of different types of treatment and there was nothing"* *"Regarding my diagnosis, I believe strongly that the doctor should have been able to refer me, or the next lot of people that it happens to, to a counsellor or to someone who will actually give you a cup of tea for a start. The diagnosis should not be delivered to people like it was to me. "If you were sick for a period of time, you could expect something like that, but it was like delivering a life sentence when you hadn't murdered anyone and weren't on trial"* *"I (also) believe that anybody who has been diagnosed with cancer should have their next appointment, as soon as possible, with a counsellor"* *"As well as the diagnosis, I was just hit with - you know when you find pancreatic cancer it's usually too late and there's not much that can be done about it. That seemed to be the general attitude. I was in hospital at the time and it just seemed to be the attitude amongst all medical professions, right down from the intern to the specialists"* *"Some people want to take part in trials as they want the latest in treatment. Could that be part of an information package with regard to options?"* *"I came away and, even in the horror of things, I said to my friend that he sounds like a very good used car salesman. So he offered me one (chemo option) with another three. I asked him what if and he quickly gave me a very short spiel of the risks attached"*	Clinical communication
*"When someone says they have this cure and I say "yes, but it's only for breast or bowel cancer", which is nothing compared to what I have.... "We need the information out there that it is advanced, unique and aggressive.... I agree with general community awareness because people need to know. I didn't know that the pancreas was so important...."* *"We need a central information point, whether its written literature or whatever, that can be referred to immediately"* *"Some people might even decide to go overseas for treatment and to try different things. So information is very important. There are many other alternatives and some of them may not be cheap"* *"Maybe if people knew what some of the symptoms were, like ovarian cancer, they would go and get checked"* *"The other suggestion is the immediate sharing of information. You hear something and you would like to assist and support that person....... I would be only too happy to volunteer my time for so many hours a week to help with that if I could"*	Public awareness

*"A few of us have had the Whipple procedure, but there are variations within the procedure itself. I know that most progress has been made in America and I'm sure there are various good reasons why there are delays in getting that information here. I wonder whether we can speed up that process or have our surgeons trained more quickly"* *"It sounds awful but it sometimes does seem to be the quality of your GP that gets the results quicker too and some of the medical profession not even knowing, so there's a lack of awareness as well"* *"I went to the doctor last week and I couldn't get in to see my regular doctor so I just saw one at the local medical centre. I told him I had had a Whipple procedure. He had never heard of a Whipple procedure and this is a doctor"* *"There seems to be a lack of awareness with GPs and also specialists even just knowing about enzymes. Some people say their doctor has never mentioned it, and I've spoken to a few people who have had a Whipple procedure"* *"My naturopath mentioned enzymes so I'm taking a supplement. That's what I was saying before -- it's either lack of information or people just say to keep eating normally, which is not the right thing to do because everyone is different and people react differently to different foods"* *"At no time was I given an option. I have heard from other people that there are different types of medication that may be more costly; however, they were never given the choice to decide to pay for the cost of more expensive medication....... I think it's up to the cancer patient to make a decision as to which treatment is more suitable for them and whether they have the means to pay for more expensive drugs not covered by Medicare or a private fund"* *"I believe that we need a coordinated group of people who are, say, oncologists and surgeons and radical people and alternatives"*	Quality of care

**Table 3:** Carers repeated issues and focal themes

**Recurring issue**	**Focal theme**
*"With the diagnosis, I wish there was some sort of way they could have done it more quickly"* *"From my wife's experience, it was 12 months or even longer.... We were backwards and forwards to doctors. It was only in the latter stages that it was diagnosed"* *"From my experience as well, that it was the detection ... and the diagnosis which was delayed which meant the disease had progressed"* *"I can only echo what the others have said. The issue, certainly in my mother's case, was no detection... it was only when the cancer had spread"* *"It just seems that early detection is the really only useful thing that can help in saving lives"* *"I totally agree with everything that everyone has said today. Early detection.... if that can all be brought in to help other people"*	Early detection
*"When the actual first diagnosis was given to us, we had an unfortunate experience as it was rather brutal. My wife was told to get her affairs in order. That's not treating the individual as a whole person. We just felt very badly let down"* *"I didn't feel that the manner of delivery was particularly warm. It's almost like some of the specialists are so experienced with what they're doing that they lose sight of the fact that for the person who's been diagnosed, it's the first time they've ever heard these words. Very often what's been told is there is actually nothing that can be done. Maybe there could be a set of words and communication could be improved a little bit?"* *"Anything we mentioned was virtually cast aside, almost contemptuously... The prognosis may be dismal, but my wife was entitled to a lot more than that. The human spirit demands more than that"* *"I think guidelines would be helpful there, taking into account the sensitivity of the individual"* *"(The) ways that the doctors come out and tell you. There have to be some guidelines that they need to go by in a situation like this"* *"I think you reach a point where you can't read anymore and you can't search the internet anymore and you just need somebody to sit next to you and talk to you and explain things to you"* *"That conversation regarding pancreatic cancer is never going to be a nice one and we have to avoid shooting the messenger there. Maybe there could be some protocol that specialists follow a little more thoroughly. No-one is ever going to want to hear it so it's never going to be well received"*	Clinical communication
*"I see a priority in education/awareness of pancreatic cancer because I've spoken to people who don't even know what it is or where it is"* *"People have got to know what pancreatic cancer is and what some of the symptoms are, and I think we all acknowledge they are very vague, but the sharpening of that focus on "this could be" or "you need to". It's only when we know about something that we'll follow up on it"* *"Pancreatic cancer may have been very familiar for an oncologist, but for the individual it isn't"* *"When my mum's diagnosis came out, I was very much in the dark. I didn't get any information"*	Public awareness

*"When my father was diagnosed, we had some trouble making a decision about whether to just go with chemotherapy or the surgical option. We were having difficulty trying to ascertain which the best way to go was and we couldn't find a lot of information on what had a better outcome or did surgery exacerbate things? So this was another issue that came up with our family... treatment options"* *"Less painful and distressing treatment"* *"I know everything is hypothetical, but you have your operation and go through all of that, but there are really not enough options for you to make decisions about what you will or won't do"*	Treatment options

#### Early Detection

All participants appeared to have a well-developed understanding of the lack of current knowledge with regard to the causes of pancreatic cancer and the important benefits of early cancer detection. Many expressed concern for the urgent need for improvements in the early detection and formal diagnosis of pancreatic cancer not only for themselves or their loved ones, but for others who may develop the disease in the future:

"I would like to see some work done in the area of symptoms and diagnostic tools so people who have got a problem can hopefully get a quicker result"

Some pointed out their low personal health locus of control with regard to detecting pancreatic cancer:

"I found out when it was too late because I had no symptoms to indicate a tumour of the pancreas "

Others offered ways that research might find solutions to the problem of asymptomatic early detection:

"I was given to understand that all cancers...are a genetic failure of some sort. A mistake is multiplied on and on and gets out of control. It's like the body has a spell-check system, putting it very simply. A word can be spelt wrong but it's still a word and it's not identified. Is there any way of looking at the body's spell-check system to see if there are any mistakes there that haven't been detected?"

#### Clinician Communication

All participants verbally expressed some alarm, many with an underlying tone of disbelief, in the insensitive nature of dialogue used by the clinicians in the provision of the pancreatic cancer diagnosis and/or management options.

"I hate him for what he said .....5 o'clock on a Friday night I'm a healthy person, I'm lively, I had a life planned to 95, and he (doctor) told me I would be dead in six to 18 months. I was stunned......"

Some felt it was extremely important to be allowed to express their own perceptions of their illness; however their clinicians had not provided this opportunity:

"Anything we mentioned was virtually cast aside, almost contemptuously... The prognosis may be dismal, but my wife was entitled to a lot more than that. The human spirit demands more than that"

One carer came to the defence of medical specialists and offered a solution to the problem:

"That conversation regarding pancreatic cancer is never going to be a nice one and we have to avoid shooting the messenger there. Maybe there could be some protocol that specialists follow a little more thoroughly"

There was general agreement in both the carer and patient groups for the pressing need for health professional communication guidelines which take into account the sensitivity of the individual affected by pancreatic cancer.

#### Public Awareness

Overall there was low confidence in the ability of available public information to provide all that was needed by people affected by PC.

" see a priority in education/awareness of pancreatic cancer because I've spoken to people who don't even know what it is or where it is"

Particular concerns with regard to public awareness of pancreatic cancer differed somewhat between the patient and carer groups. Patients highlighted the need for reliable information on effective treatments:

"Some people might even decide to go overseas for treatment and to try different things. So information is very important. There are many other alternatives and some of them may not be cheap"

One patient believed their illness was not taken as serious as it should have been by others in the community because of a lack of general awareness. Other patients felt that the available information on PC needed to be made more easily accessible than it currently was:

"We need a central information point, whether its written literature or whatever, that can be referred to immediately"

There was general consensus among carers for the need for public information that had the potential to save lives in the future:

"People have got to know what pancreatic cancer is and what some of the symptoms are, and I think we all acknowledge they are very vague, but the sharpening of that focus on "this could be" or "you need to". It's only when we know about something that we'll follow up on it"

#### Quality of Care

The patient group was united in their thinking on the need for improvements in the standard and consistency of clinical care and understanding of PC:

"A few of us have had the Whipple procedure, but there are variations within the procedure itself. I know that most progress has been made in America and I'm sure there are various good reasons why there are delays in getting that information here. I wonder whether we can speed up that process or have our surgeons trained more quickly"

Health professional team coordination was also identified as being important:

"I believe that we need a coordinated group of people who are, say, oncologists and surgeons and radical people and alternatives"

#### Treatment Options

Carers voiced the need for improved and additional treatment options for PC:

"I know everything is hypothetical, but you have your operation and go through all of that, but there are really not enough options for you to make decisions about what you will or won't do"

## Study Strengths and Limitations

To guarantee an accurate summary of the group discussion and give an assurance that meaning had been correctly captured, all topics raised were summarised during the discussions to give participants an opportunity to immediately confirm or alter. This approach adds strength to the study's internal reliability and validity.

While the qualitative methodology used in this study is likely to be the most appropriate to gather information from this highly vulnerable group where there are only small numbers available to participate in research at any one time, it has several limitations. The primary study limitation is related to the small number of participants and the convenience nature of the sampling strategy which significantly limits the ability to broadly generalise the findings. Furthermore, the small sample size could mean that the full breadth of concerns of PC consumers may not have been adequately captured. There is a critical need for additional research to further validate both our findings and the utility of a telephone-based approach to focussed discussions.

## Discussion

The ethical and practical issues of inquiries with people with advanced cancer need to be carefully planned and appropriate safeguards implemented. Facilitated telephone discussion groups supported by trained counsellors allowed us to reliably and suitably canvass the views of a highly vulnerable consumer group. The approach provided PC patients and carers the opportunity to become involved in the research priority-setting process in a manner that created minimal disruption to their lives, and took into account varying levels of pain, mobility and psychological distress. It was a welcomed opportunity for these groups to express their needs in terms of research.

One of the difficulties of engaging PC consumers in research is the relatively short time between disease diagnosis and death. Time-economy was a fundamental consideration in the planning and implementation of the current research. PC develops and spreads silently, and closes in swiftly. Engaging with people who suffer from this highly lethal, time limited disease is challenging as there is often only a small window of opportunity available. Capturing those affected early via established mechanisms such as targeted projects, patient advice lines or clinician referrals are likely to be the best avenues to provide those interested with the opportunity for research involvement.

Research areas perceived by consumers to aid progress in PC research in Australia have been identified via discreet focussed discussions. Based on the direct experiences of PC consumers, five themes have been identified as being important subjects for further research. Early detection, clinician communication, public awareness, quality of care and improved treatment options are concluded to be priorities for improving wellbeing and survival in PC. The lack of existing similar investigations indicates the difficulty of the task of sampling and investigating the priorities held by PC consumers.

A common criticism of consumer involvement in expert domains is the potential to obtain individual extreme perspectives. While we found clear areas of accordance and difference between the priorities of PC consumers and researchers, the identified consumer priorities are not beyond the realms of reality or action; this supports the growing argument for more involvement of consumers in health and medical research generally.

We found general agreement between the research priorities of consumers and those of researchers who took part in our consensus process in the areas of early detection, quality of care and the need to identify optimal treatments. Priority areas that differed between the two groups included the identification of prognostic markers; the most common disease and treatment related sequelae of PC on patients and their carers and strategies for managing them; patient-doctor communication and consumer information on pancreatic cancer.

The importance of early diagnosis and more effective treatment for pancreatic cancer, including options for defining patients at high risk of pancreatic cancer, and developing methods for detecting pancreatic tumours at an early stage is starting to receive increasing attention from a number of researchers globally [20-22] although it lags well behind similar research on other cancer types such as breast cancer. In addition, there is a growing but relatively early focus on the biology of PC which is hoped will lead to multiple potential therapeutic options by directing treatment at these new biologic targets [23,24].

Sensitive information is normally conveyed to patients and carers by clinicians through verbal communication. Just how this is done varies considerably from clinician to clinician [22]. The general need for further research into the best methods for communicating serious health problems effectively and respectfully has been recognised by some researchers, although effective solutions and noteworthy improvements remain elusive [26-28]. Guidelines for effectively communicating bad news to cancer patients have been developed and widely distributed to oncologists [29,30]. However, a review of the effectiveness of these guidelines has found that they may not reflect the complexities of patient-oncologist interactions [31]. Other studies have found that the ability of the cancer patient to recall what the oncologist has said is influenced by prognosis, with patients with a poorer prognosis recalling significantly less [32]. The findings of poor patient acceptability, didactic approach and lack of wider family communication have led to a recent inquiry to call for an urgent revision of existing terminal prognosis communication protocols and practices taught in medical school training [33].

More widespread and easily accessed public information on pancreatic cancer has been recognised by a handful of existing overseas patient support organisations [34,35] as critical in raising awareness of possible symptoms, providing a reliable avenue for accurate and current treatment and management information, and linking people to answers and others affected by the disease. Unlike studies that exist for many other cancer types [36-39] we could find no formal inquiry investigating the best approaches, true level of availability and effect of relevant information resources on PC consumers.

The growing impetus to better address PC, including the results of this first consumer inquiry, may help increase focus by, and pressure on, health and medical research funding organisations to further discriminate in favour of this under prioritised and underfunded health problem.

## Conclusion

This research gives us the first understanding of the role that consumers with a known limited life expectancy can play in identifying research that is responsive to community needs and priorities when suitably planned and implemented. Research has traditionally been controlled by scientists to the exclusion of a societal perspective. Investigating the issues faced by PC patients and carers goes a long way towards understanding how science might best 'fit' with their needs, contexts and expectations. The research also provides a much better understanding of the stark realities of the disease for those affected.

## Competing interests

The authors declare that they have no competing interests.

## Authors' contributions

CS undertook the data analysis and literature review, and was the primary author of the paper; HG coordinated the project, collected the data, contributed preliminary input to the paper; and continues to oversee the associated program; MR undertook the data analysis and contributed to the development, drafting and editing of the paper; JM providing valuable project assistance and comments and suggestions during the preparation of the paper.
